# Impact of the Electrode Material on the Performance of Light-Emitting Electrochemical Cells

**DOI:** 10.1021/acsami.4c18009

**Published:** 2025-01-10

**Authors:** Anton Kirch, So-Ra Park, Joan Ràfols-Ribé, Johannes A. Kassel, Xiaoying Zhang, Shi Tang, Christian Larsen, Ludvig Edman

**Affiliations:** † The Organic Photonics and Electronics Group, Department of Physics, Umeå University, SE-90187 Umeå, Sweden; ‡ LunaLEC AB, Umeå University, SE-90187 Umeå, Sweden; § Max Planck Institute for the Physics of Complex Systems, Nöthnitzer Straße 38, 01187 Dresden, Germany; ∥ Wallenberg Initiative Materials Science for Sustainability, Department of Physics, Umeå University, SE-90187 Umeå, Sweden

**Keywords:** light-emitting electrochemical cells, electric double layers, exciton generation profile, electrode work function, surface plasmon polaritons, optical modeling

## Abstract

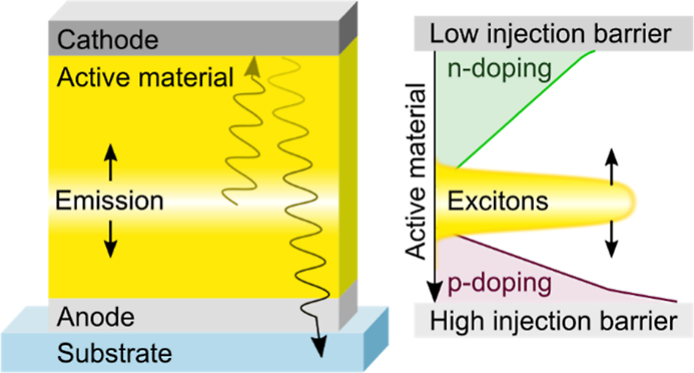

Light-emitting electrochemical cells (LECs) are promising candidates for fully solution-processed lighting applications because they can comprise a single active-material layer and air-stable electrodes. While their performance is often claimed to be independent of the electrode material selection due to the in situ formation of electric double layers (EDLs), we demonstrate conceptually and experimentally that this understanding needs to be modified. Specifically, the exciton generation zone is observed to be affected by the electrode work function. We rationalize this finding by proposing that the ion concentration in the injection-facilitating EDLs depends on the offset between the electrode work function and the respective semiconductor orbital, which in turn influences the number of ions available for electrochemical doping and hence shifts the exciton generation zone. Further, we investigate the effects of the electrode selection on exciton losses to surface plasmon polaritons and discuss the impact of cavity effects on the exciton density. We conclude by showing that we can replicate the measured luminance transients by an optical model which considers these electrode-dependent effects. As such, our findings provide rational design criteria considering the electrode materials, the active-material thickness, and its composition in concert to achieve optimum LEC performance.

## Introduction

Organic optoelectronic devices can be fabricated by low-energy and resource-efficient solution-based methods using nontoxic materials, which is key to reducing their carbon and waste footprint.^1−3^ Among others, solution-processed light-emitting diodes (OLEDs),^4^ wavelength and oxygen sensors,^5,6^ photoluminescent tags (PLTs),^7^ photovoltaics (OPVs),^8^ and electrochemical transistors (OECTs)^9^ have been demonstrated. Light-emitting electrochemical cells (LECs) are particularly appealing in this context, as their entire device structure, i.e. the single active-material layer and the two air-stable electrodes, can be fabricated under ambient conditions^10,11^ using nontoxic solvents.^12^ From a sustainability perspective, this holds an advantage over advanced and more efficient multilayer p–i–n OLEDs, which are usually fabricated by energy-intense high vacuum processing.^13,14^

In LECs, the single-layer active material (AM) is sandwiched between two electrodes and consists of an emissive organic semiconductor (OSC) and mobile ions. Under applied bias, the mobile ions redistribute and form electric double layers (EDLs) at the electrode/AM interfaces, causing a low injection resistance for charge carriers into the OSC. The remaining mobile ions drift according to the local electric field and electrically compensate for the space charge generated by the injected electrons and holes, a process called electrochemical (EC) doping. Over time, these n-type and p-type doped regions grow from the cathode and anode, respectively, lowering the transport resistance within the AM. Electrons and holes meet between the doped regions and generate excitons, which are intended to decay radiatively under the emission of photons. This exciton generation zone (EGZ) may also be referred to as emission zone,^15^ p–i–n junction,^16^ or p–n junction,^17^ but since a focal point of this work is on determining and discussing the exciton generation profile, we use the term EGZ.

The in situ forming doping profiles and thus the EGZ dynamics depend on the LEC driving conditions, the composition of the AM, and the ion and polaron mobility.^15^ The dynamic formation of a self-organized doping structure in a single-layer AM stands in contrast to the as-fabricated multilayer architecture of a p–i–n OLED, where individual, molecularly doped layers enable charge-carrier injection, transport, and recombination at predefined and optimized positions.^18−20^

As for the electrode materials, it is frequently implied that the formation of EDLs renders the LEC performance independent of the electrode material selection.^21−23^ This is a reason why LECs are particularly suitable for ambient-air printing. It has been demonstrated, however, that extrinsic degradation related to the electrode material can have a detrimental influence on LEC stability.^24^ Notably, if the oxidation (reduction) potential of the positive anode (negative cathode) is positioned at a less positive (negative) potential than the p-type (n-type) doping potential of the OSC, the preferred electrochemical reaction at the anode (cathode) is oxidation (reduction) of the electrode instead of p-type (n-type) doping of the OSC. This undesired scenario leads to electrode degradation and premature device failure.^25^ Similarly, it has been shown that other compounds in proximity to the electrode/AM interfaces, e.g. ion transporters, O_2_ or H_2_O impurities,^26^ can also cause or be part of electrochemical side reactions.^27,28^

In this study, we show that the choice of the electrode material can have a strong additional intrinsic influence on the in situ forming doping structure in the AM and the properties of the optical cavity, two factors that determine the LEC performance. We investigate the impact of the cathode selection (Al, Ag, or Ca) and the AM thickness on the position of the EGZ, the excitonic coupling to surface plasmon polaritons (SPPs), and the Purcell factor, while keeping a common AM composition and indium tin oxide (ITO) anode. We conclude by qualitatively replicating the measured electrode-induced luminance changes by an optical simulation.

## Results and Discussion

### LEC Performance

**Figure 1:**
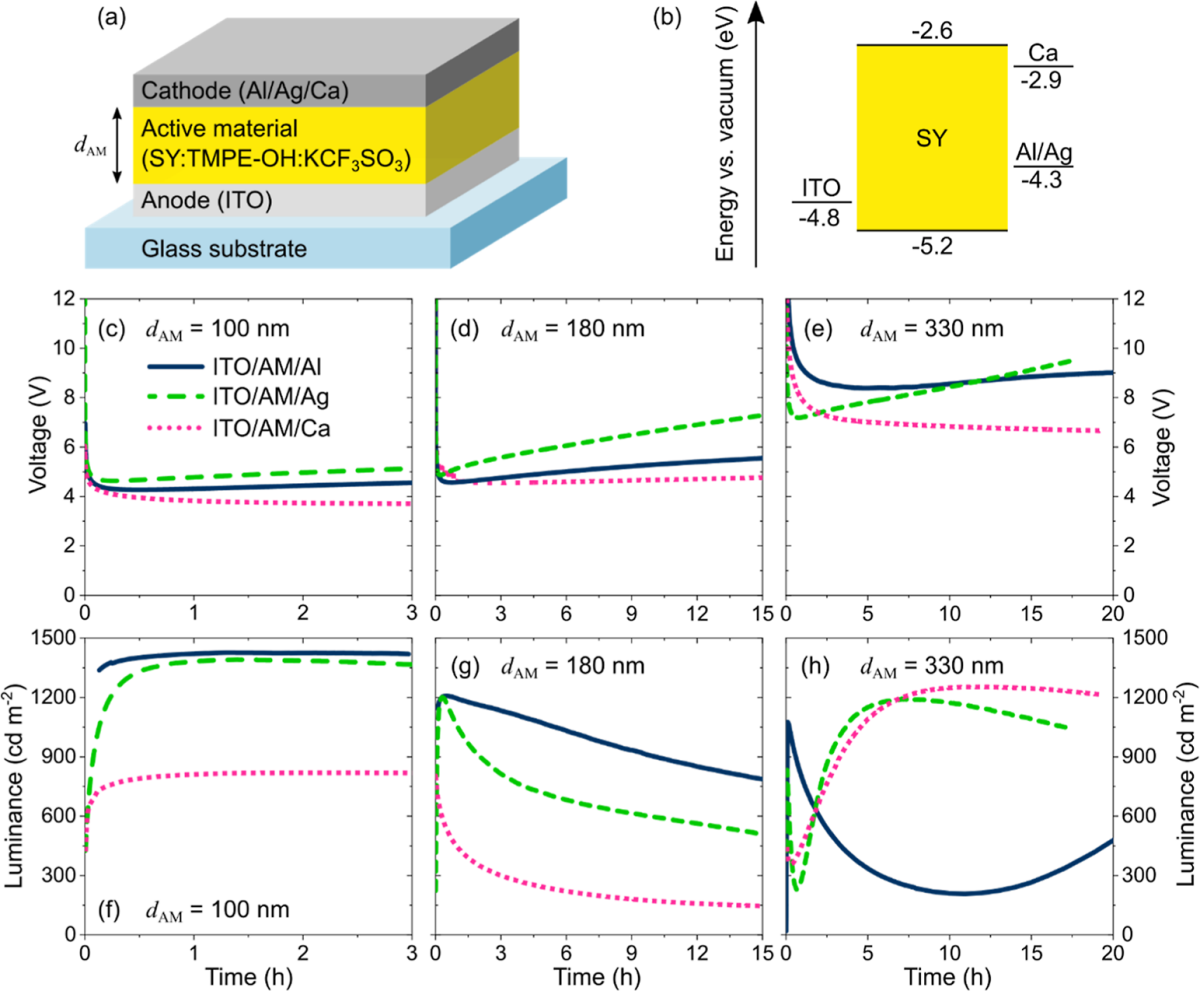
(a) LEC structure with the active material (AM) sandwiched between a reflective top cathode and a transparent bottom ITO anode on a glass substrate. The investigated cathode materials are Al, Ag, and Ca, while the investigated AM thicknesses (*d*_AM_) are 100, 180, and 330 nm. The encapsulation barrier is omitted for clarity. (b) Energy level diagram showing the work function of the electrodes^24^ and the HOMO/LUMO levels of Super Yellow (SY).^29^ (c–h) Temporal evolution of (c–e) voltage and (f–h) forward luminance of LECs comprising different cathode materials (see label in c) for different *d*_AM_ under constant current-density operation at 25 mA cm^–2^.

Figure 1a displays the bottom-emitting LEC structure used throughout this work. It comprises a glass substrate, a transparent indium tin oxide anode (ITO, thickness = 145 nm), an active material (AM) of thickness *d*_AM_, and a reflective cathode (Al, Ag, or Ca, thickness = 100 nm). The AM consists of the electroluminescent semiconducting polymer Super Yellow (SY), the ion-transporting compound TMPE–OH, and the salt KCF_3_SO_3_, in a mass ratio of 1:0.1:0.03.^30^ Three different thicknesses for the AM are investigated: *d*_AM_ = 100, 180, and 330 nm. Note that, although the fabrication parameters are kept constant, the thickest *d*_AM_ is found to vary between 320 and 340 nm for different samples. The *d*_AM_ variation of a single AM film is invariably below 5 nm, see Experimental section for details. The device is protected from oxygen- and water-induced degradation by an encapsulation glass that is attached by epoxy glue on top of the reflective cathode (not shown in Figure 1a). The Ca cathode consists of 20 nm of Ca in contact with the AM, and 80 nm of Al to protect Ca from contamination during encapsulation.

Figure 1b presents the work function (WF) of the anode and cathode materials^24^ as well as the lowest unoccupied molecular orbital (LUMO) and the highest occupied molecular orbital (HOMO) levels of SY.^29^ Note that the energy difference between the WF of the cathode (anode) and the LUMO (HOMO) defines the height of the electron (hole) injection barrier. For the three investigated LEC structures, the electron injection barrier varies between 1.7 eV (for Ag and Al) and 0.3 eV (for Ca), while the hole injection barrier at the ITO anode remains unchanged at 0.4 eV.

Figure 1c–h show the temporal evolution of the voltage (c–e) and the forward luminance (f–h) of the nine different LECs when driven at a constant current density of 25 mA cm^–2^. For all devices, the driving voltage decreases, and the luminance increases during the initial operation, cf. Supporting Information, Figure S1, for a close-up of the initial operation. These two LEC-characteristic observations imply that all devices form injection-enabling EDLs and develop a doping structure that facilitates charge-carrier transport and recombination by in situ EC doping.^17^

As expected, the minimum voltage increases with increasing *d*_AM_, because the EGZ, i.e. the most resistive part of the LEC sporting the lowest doping level, widens with increasing *d*_AM_.^31^ Similarly, the time required to reach the minimum voltage is also increasing with *d*_AM_ (note the different time scales in Figures 1c–f), as the ions building doped layers need to migrate longer distances.

We further find that the time required to reach the minimum voltage is dependent on the cathode selection, which suggests that the magnitude of undesired, conductivity-degrading side reactions depends on the electrode material. In this context, we note that the electrolyte TMPE–OH/KCF_3_SO_3_ has been demonstrated to exhibit a reduction potential in the proximity of the LUMO level of SY. This implies that side reactions can take place in parallel with the preferred EC n-type doping of SY. The observation that the Ag-cathode, and to a lesser extent the Al-cathode, devices exhibit a faster increase in voltage with time thus suggests that Ag, and to a lesser extent Al, “catalyzes” such a conductivity-degrading side reaction.

More surprising is that the cathode selection has such a strong influence on the luminance transients, while all samples comprise the same AM. In the following, we will therefore investigate the impact of the cathode selection and *d*_AM_ on the EGZ, the coupling of excitons to surface plasmon polaritons, and the properties of the optical cavity.

### Center of the Exciton Generation Zone (CEG)

As for all thin-film electroluminescent (EL) devices, the position of the EGZ can strongly influence the light generation and outcoupling efficiency in LECs, which determines the perceived luminance.^19,32^ Since the formation of the doping structure is a dynamic process in LECs, the temporal evolution of the EGZ is crucial information to understand the luminance transients encountered in Figure 1f–h. We determine the center of the exciton generation zone (CEG) for the nine different LECs by measuring their angle-dependent emission spectra with a spectro-goniometer. These data are compared to the angle-dependent emission spectra generated by an optical model of the LEC using the commercial software Setfos. The model assumes a Gaussian-shaped exciton generation profile *G*(*x*) in (m^–3^ s^–1^) with the center position CEG and the full width at half-maximum FWHM_EG_ as fitting parameters, which are optimized via an estimation algorithm. The best-fitting estimator CEG, i.e. the CEG that minimizes the mean squared error between measurement and simulation, discloses where the center of the EGZ is located in the AM, see Experimental section and refs (15 and 33) for details.

**Figure 2:**
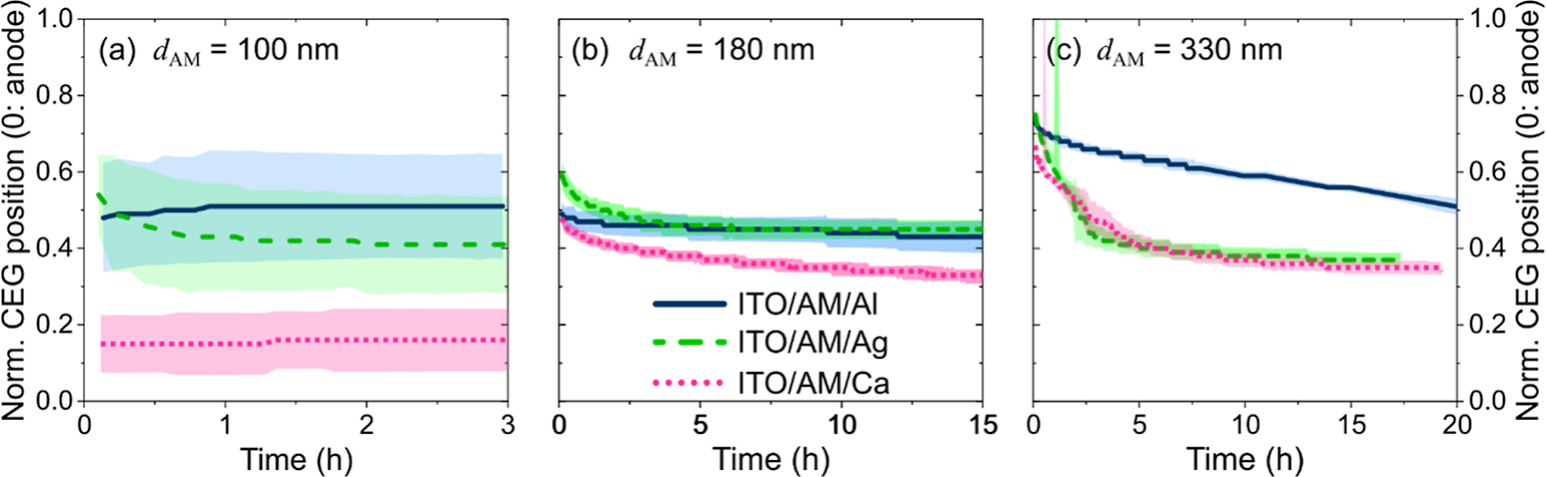
Temporal evolution of the center of the exciton generation zone (CEG) in LECs with different cathode materials (see legend in b) and the different values of *d*_AM_ (a–c). The CEG position is normalized to *d*_AM_ with 0.0 denoting the anodic and 1.0 the cathodic interface. The lines indicate the best fit between the simulated and measured angle-dependent emission spectra. The shaded areas represent the confidence intervals of the estimation, see details in the Experimental section.

Figure 2 displays the derived temporal evolution of the CEG position normalized to *d*_AM_, with 0.0 corresponding to the anodic and 1.0 to the cathodic interface. The shaded areas indicate the confidence intervals of the estimation algorithm, see Experimental section for details. Since cavity effects are less significant for films that are much thinner than the SY emission wavelength (peak emission at about 550 nm), i.e. far from the wavelength interference condition, the confidence intervals are larger for thinner devices.

Figure 2a shows that the CEGs for the Al- and Ag-cathode LECs (*d*_AM_ = 100 nm) stabilize within about 1 h close to the center of the AM. The CEG for the Ca-cathode LEC, however, is significantly displaced toward the anode (dotted magenta line). A similar trend is observed for *d*_AM_ = 180 nm in Figure 2b, although the relative difference between the CEGs is smaller. For the two thicker configurations in Figures 2b,c, the EGZs form initially closer to the cathode and subsequently migrate toward the center, which indicates that the anion mobility is smaller than the cation mobility.^15^ Notably, the stabilization process of the CEG takes longer for thicker films, which is why the time scales (*x*-axes) are adjusted for different *d*_AM_. As introduced above, the Ag and, to a lesser extent, the Al cathodes may catalyze a side reaction at the cathode/AM interface. Over time, we suspect this degradation will induce additional shifts to the obtained CEG transients, which are hard to quantify. Therefore, the long-term CEG assessment, especially for the thick devices, may be corrupted.

A key question is now why the thin Ca-cathode LEC features a displaced CEG compared to the corresponding Al and Ag devices. As the CEG is located between p- and n-doped regions in the AM, the observation implies that the doping profiles are shifted when the cathode material is changed from Ca to Al or Ag. In this context, it is important to note that the electron injection barrier is much smaller with Ca as the cathode (0.3 eV) than with Al or Ag (1.7 eV), cf. Figure 1b. In a running device, this reduced injection barrier causes a smaller potential drop across the EDL, a lower cation concentration in the cathodic EDL,^34^ and thus increases the number of cations that remain available for EC n-doping. We propose that these excess cations produce a net shift of the doping structure and the CEG toward the anode.

To rationalize this hypothesis, one can estimate the number of ions that are consumed in an EDL by treating the EDL as a parallel-plate capacitor. Assuming a plate (charge) separation of 0.5 nm, one can estimate that about 7% of all cations available in the AM form the EDL at the AM/Al and AM/Ag interface for *d*_AM_ = 100 nm, while only about 1% of them are required for the AM/Ca interface, see Supporting Information Section 2 for the detailed estimation. This reduction in EDL-consumed cations for the Ca-cathode LEC increases the maximum attainable n-doping level. Thus, it decreases the driving voltage under constant-current conditions and causes a shift of the CEG toward the anode. This effect would be more pronounced for a small *d*_AM_, as the number of ions in the EDL scales with the surface area of the device (unaffected by *d*_AM_) while the total number of available ions increases with the volume of the AM (linearly with *d*_AM_). This reasoning can explain both the encountered CEG shift for the thin Ca-cathode LEC, as well as its lower observed driving voltage, cf. Figure 2.

The impact of this EDL-induced CEG shift depends on the actual number of mobile ions in the AM. If we assume that all salt complexes that are experimentally incorporated into the AM dissociate into mobile ions, i.e. contribute to either the EDL formation or EC doping, and that Al or Ag (Ca) capture merely 7% (1%) of the cations in the cathodic EDLs, 93% (99%) of the cations remain available for EC doping and the overall effect on the CEG and the driving voltage would be minor. However, previous studies propose that a significant share of the ions does not contribute to either of the two processes.^35,36^ This would increase the ratio of mobile ions consumed by the EDLs and make the doping profiles more asymmetric. We are currently working on quantifying how many ions contribute to EC doping by conductivity measurements. If we can confirm that this number is indeed significantly lower than the experimentally introduced salt density, it would build a strong case for the reasoning above and substantially refine the understanding of LEC physics. The reasoning that adjusted injection barriers can significantly alter the doping profiles in LECs and influence the number of ions attainable for doping could pose a handle to reduce the required salt concentration and thus contain the detrimental effect of exciton-polaron quenching.^16^

### Surface Plasmon Polariton (SPP) Losses

The electrode/AM interface can also impact the exciton density via radiative near-field coupling of excitons to surface plasmon polaritons (SPPs). SPPs travel along the electrode/AM interface and are predominantly excited if the excitonic dipole in the AM is vertically oriented.^19,32,37^ The radiative nature of SPP coupling causes an increased effective radiative exciton decay rate k*_r_(*x*) close to the electrode and therefore depopulates the exciton manifold. While polariton losses in OLEDs are usually contained by an appropriate transport layer design^19^ or can be utilized to enhance the device lifetime,^38^ we will show that they are a major loss mechanism for practical thin-film LECs (*d*_AM_ ≈ 100 nm) if the EGZ is close to an electrode.

To quantify the exciton losses to SPPs and their dependence on the electrode material selection, we use the same optical simulation as above for a slightly adapted stack model. It merely comprises an AM layer on top of a 100 nm thick electrode (either Al, Ag, Ca, or ITO). The AM thickness is chosen to be infinite to exclude further cavity influences (as discussed in the next section). The Ca electrode comprises 20 nm of Ca and 80 nm of Al, as introduced above for the Ca-cathode LECs. The exciton generation profile *G*(*x*) is modeled as a delta distribution, using the measured SY anisotropy coefficient *a* = 0.05.^39^ The anisotropy coefficient *a* describes the relative contribution of out-of-plane dipoles to the forward luminance and thus defines the average exciton dipole alignment to the stack normal. It takes values between 0 (horizontal) and 1 (vertical orientation).^40^

**Figure 3:**
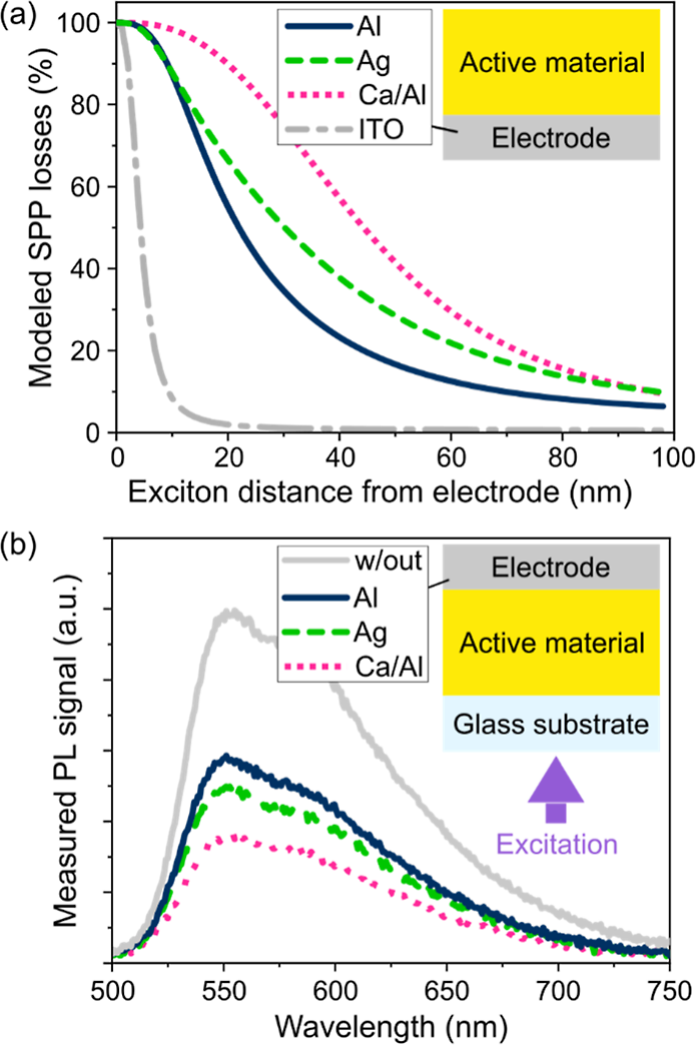
(a) Simulated SPP losses of a delta-shaped exciton generation profile as a function of its distance from the electrode surface for the four different electrode materials and an anisotropy coefficient *a* = 0.05. (b) Photoluminescence spectra of a 25 nm thin AM film deposited on a quartz-glass substrate dependent on the top electrode selection as presented in the inset.

Figure 3a presents the simulated ratio of excitons coupling to SPP modes as a function of the spatial separation between the exciton generation profile and the electrode/AM interface for the four employed electrode materials. We find that SPP losses decrease monotonously with increasing exciton-electrode separation for all four electrode materials, which is in line with the established understanding of SPP loss modes for horizontally aligned dipoles.^41−43^ According to the simulation, Ca is by far the strongest exciton quencher of the investigated electrode materials, followed by Ag, Al, and ITO.

Figure 3b exemplifies this dependency of SPP-induced exciton losses on the electrode material in a photoluminescence (PL) experiment. Here, we deposit an AM film (same composition as above) with *d*_AM_ = 25 nm on a quartz glass substrate. On top of that film, one of the indicated cathode materials is evaporated. The AM is excited by UV light (λ_peak_ = 450 nm) through the glass substrate, and the resulting PL signal is recorded in an integrating sphere. A sample without an electrode is measured for comparison. The PL intensity is most severely reduced by the Ca electrode, followed by Ag and Al. The highest PL intensity is recorded for the reference sample with no electrode deposited on the AM film. While we investigate undoped films in this experiment, which stands in contrast to an LEC where the optical properties of the OSC are changed by doping,^44^ these findings illustrate the magnitude of SPP losses and are in line with the model prediction in Figure 3a.

### Purcell Factor and Exciton Density

The exciton dynamics within the AM of an LEC are significantly influenced by the optical environment, a characteristic shared by all sandwich-type EL devices. When describing the properties of the stack’s optical cavity, the complex refractive index (*n* and *k* values) of the electrode material influences the reflectivity and phase-shifting properties of the electrode/AM interfaces. The resonance between excitons and reflected photons, together with the excitonic coupling to SPPs, produces a local Purcell factor *F*(*x*) in the AM^45−47^ which alters the natural radiative decay rate *k*_r,0_, rendering it a position-dependent effective radiative decay rate k*_r_(*x*).^19,37^
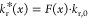
1

To discuss the influence of the cathode material on the steady-state exciton density ρ(*x*), which links to the number of photons generated in the device, we compare the simulated k*_r_(*x*) for the investigated LECs. This assessment is based on a transfer-matrix algorithm that can be solved in Setfos. It takes the optical properties of the cavity and the exciton generation profile *G*(*x*) to calculate k*_r_(*x*) and ρ(*x*). To concisely illustrate the most important effects, we do not use the actual, time-dependent CEGs obtained from Figure 2, but assume a common, centered second-order super-Gaussian *G*(*x*) with FWHM_EG_ = 0.2·*d*_AM_. This is a Gaussian function with a squared exponent. It yields a flattened center which was found to be a reasonable estimate for real exciton generation profiles.^39^ A nonradiative decay rate *k*_nr_ = 2 × 10^8^ s^–1^, a natural radiative decay rate *k*_r,0_ = 3 × 10^8^ s^–1^, and a drive current density of 25 mA cm^–2^ are used in the simulation.^48^ Note that this model is purely optical and does not consider Förster-type losses to the electrodes, which nonradiatively depopulate the exciton manifold close to (<25 nm) the electrodes and thereby exacerbate electrode-induced losses.^49^ For additional details on the modeling, see the Experimental section.

Under excitonic steady-state operation and neglecting exciton movement and interaction, the exciton generation *G*(*x*) in (m^–3^ s^–1^) equals the exciton decay *D*(*x*), which is the product of the exciton density ρ(*x*) in (m^–3^) and the exciton decay rate *k*(*x*) in (s^–1^). The latter is specified by its effective radiative and nonradiative components, k*_r_(*x*) and *k*_nr_, respectively.
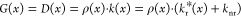
2

**Figure 4:**
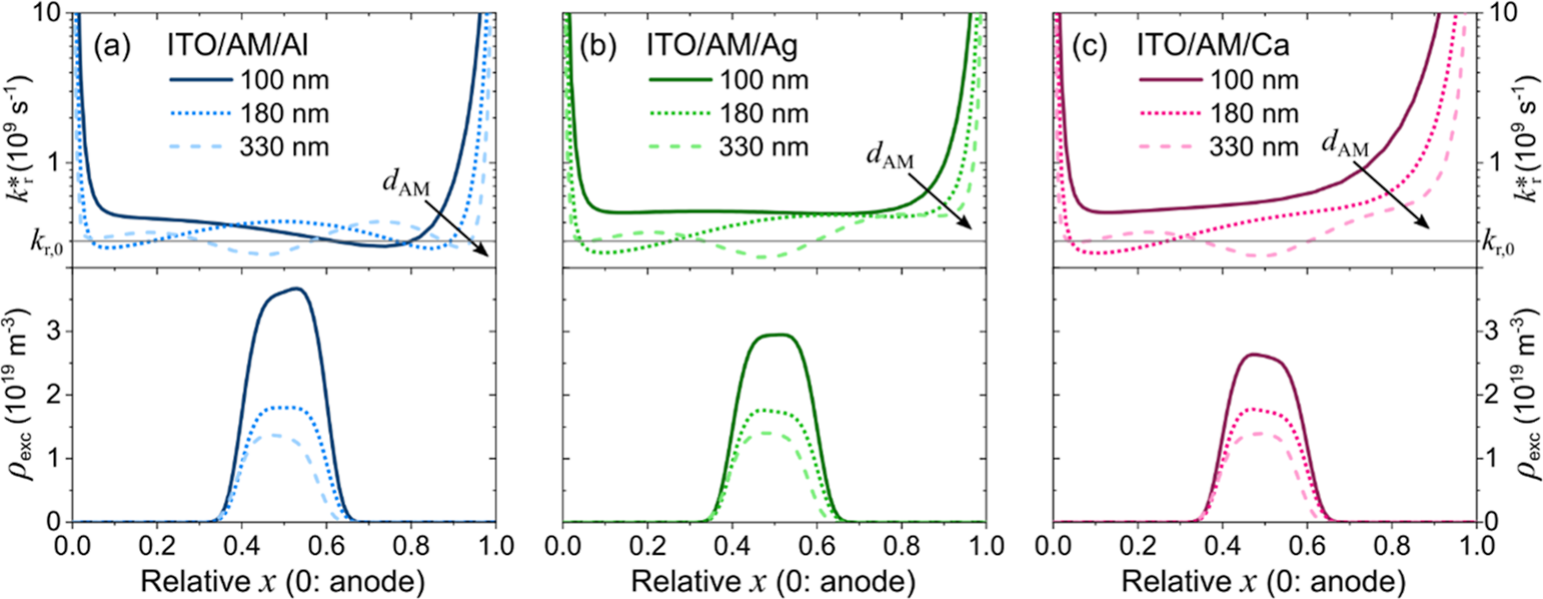
Simulated effective radiative exciton decay rate k*_r_(*x*) (upper panel) and the resulting exciton density ρ(*x*) (lower panel) as a function of the relative position *x* in the AM for (a–c) the three different cathode materials and for the three values of *d*_AM_. The simulation was performed with a common centered second-order super-Gaussian exciton generation profile *G*(*x*) at a current density of 25 mA cm^–2^.

Figure 4 presents the simulated values for k*_r_(*x*) in the upper panel and the resulting ρ(*x*) in the lower panel as a function of the relative interelectrode position *x* for the nine different LEC stacks. Note that the anode (ITO) interface is located at *x* = 0.0, the cathode interface at *x* = 1.0, and that the constant relative width of *G*(*x*) (FWHM_EG_ = 0.2·*d*_AM_) results in an absolute decrease of ρ(*x*) with increasing *d*_AM_.

The influence range of the electrode-induced SPP losses on k*_r_(*x*) is clearly visible in the upper panel of Figure 4. It is highest for the Ca cathode, lower for Ag and Al, and smallest for ITO, in line with Figure 3. The increasing k*_r_(*x*) results in a decreasing exciton density when going from Al, over Ag, to Ca for *d*_AM_ = 100 nm, cf. lower panel in Figure 4a–c, and a tilt of ρ(*x*) corresponding to the respective slope of k*_r_(*x*) around *x* ≈ 0.5. It is important to note that the investigated AM features predominantly horizontally aligned dipoles (*a* = 0.05). Here, the in-plane wave vector contributions are small and the coupling to SPP modes is only moderate.^19,49,50^ For the case of isotropic or even vertical emitter dipole orientation, the impact of SPP losses on k*_r_(*x*) is even more significant, as exemplified in the Supporting Information, Figure S2, and refs (51 and 52).

With increasing *d*_AM_, interference effects, perceivable by the undulating k*_r_(*x*), dominate the center of the device, as the absolute distance between excitons and electrodes increases and the optical thickness of the device cavity approaches the emission wavelength of SY. While the SPP influence changes significantly between the cathode materials close to the cathode (*x* close to 1), these undulating interference patterns remain relatively stable for thick films. This is most apparent for *d*_AM_ = 330 nm, where k*_r_(*x*) for *x* < 0.7 looks very similar for all investigated cathode materials, cf. upper panel in Figure 4. Hence, the resulting ρ(*x*) does not significantly differ between the investigated cathode materials for high *d*_AM_, cf. lower panel in Figure 4.

We can conclude that thick devices, which are more fit for printing applications, are less susceptible to the choice of the electrode material if the EGZ is located well away from an electrode. Since the SPP losses close to a cathode/AM interface induce the biggest deviations in k*_r_(*x*) between the investigated cathode materials, it is instead the interference pattern that determines the preferred location for the EGZ. The thinner the device, the stronger the impact of the electrode-dependent coupling to SPP modes. For thin films (*d*_AM_ = 100 nm), it becomes more important to choose a weak quencher, e.g. ITO, Al, or Ag, as an electrode material.

### Luminance Modeling

**Figure 5:**
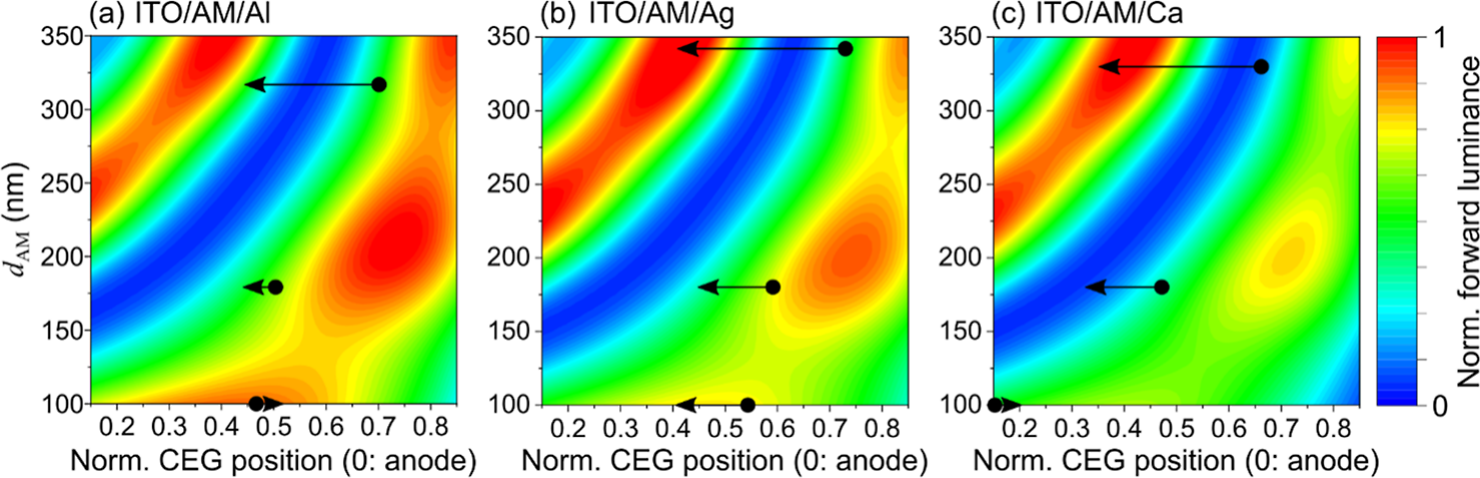
Simulated forward luminance as a function of the CEG position (*x*-axis) and *d*_AM_ (*y*-axis) for (a–c) the three investigated cathode materials. The arrows indicate the measured transients of the CEG during LEC operation.

We conclude our investigation by calculating the outcoupled forward luminance of the LEC model in Setfos depending on the three investigated cathode materials. Eventually, we compare the simulated to the experimental luminance data in Figure 1f–h. Again, this is a purely optical assessment that does not include exciton–exciton interactions or polaron quenching, i.e. it assumes a constant nonradiative exciton decay rate *k*_nr_ = 2 × 10^8^ s^–1^ and no Förster energy-transfer rate. It hence overestimates the share of excitons that decay radiatively, which is why we present the simulated luminance values only qualitatively. Also, it does not take into account the Förster-induced exciton quenching close to the electrodes, which is why the CEG position in Figure 5 is only considered for an interval 0.15 ≤ *x* ≤ 0.85.^49^ For this simulation, the exciton generation profile *G*(*x*) is assumed as a delta function to reduce computational effort, the anisotropy factor is set to *a* = 0.05, and the refractive index of SY is used for the AM, see Experimental section for details.

Figure 5 presents the simulated forward luminance of the LEC stack as a function of the CEG normalized to *d*_AM_ (*x*-axis) and the *d*_AM_ (*y*-axis) for the three different cathode materials. These contour plots summarize the impact of the discussed interference and electrode effects and provide straightforward design criteria for optimized LEC luminance. We find that the strongest deviation between the cathode materials can be seen in the bottom and right areas, where the CEG is located closest to the cathode and *d*_AM_ is small. Moreover, we find that the lowest luminance in this bottom-right area is obtained for the Ca cathode, which is in agreement with Figures 3 and 4, as Ca features the strongest SPP quenching. This finding aligns with the discussion about the impact of the electrode material on k*_r_(*x*) in the last section: Only for thin devices and close to the electrode, k*_r_(*x*) is significantly dependent on the electrode material.

To connect these contour plots to the measured luminance data in Figure 1f–h, the derived evolution of the CEGs (c.f. Figure 2) for the nine measured devices are indicated as black arrows. We find that the simulated luminance transients qualitatively reproduce the measured transients in Figure 1f–h. This means that the optical properties of the LEC cavity, depending on the cathode material and *d*_AM_, and the CEG transients are thus the origin of the differing experimental forward luminance data. This is best illustrated for the thickest devices (*d*_AM_ ≈ 330 nm), which start at intermediate luminance, cross a luminance valley, and finally reach almost their potential luminance maximum, reproducing the encountered luminance undulations in Figure 1h. For the two thinner configurations, the Ca device is expected to operate in an area of lower forward luminance, as excitons couple more heavily to SPP modes, cf. Figure 4c, which aligns with the measurement displayed in Figure 1f–g.

We can conclude that, according to Figure 5, controlling the electrode materials, the CEG, and *d*_AM_ in concert, based on optical modeling, seems a viable way to find the conditions for maximum LEC forward luminance.

## Conclusion

In this work, we investigate the influence of the electrode material selection on the performance of a common three-layer LEC stack by combined measurements and simulations. We fabricate nine different LEC configurations by systematically varying the cathode material (Al, Ag, and Ca) and the thickness of the active material (between 100 and 330 nm) and find that both parameters heavily influence the LEC performance. Through spectro-goniometer measurements, we study the transients of their exciton generation zones and observe a shift toward the anode for thin films when using a Ca cathode. To explain this observation, we propose that the number of cations forming the cathodic EDL influences the number of cations that remain available for electrochemical doping. In that manner, the exciton generation zone and the device resistance are directly affected by the difference between the cathode WF and the semiconductor LUMO, i.e. the electron injection barrier.

Apart from the impact on the doping structure, we investigate how the electrode material influences exciton losses to surface plasmon polaritons. They are shown to be a dominating and heavily material-dependent loss channel for common thin-film LECs, even if the mean emitter dipole orientation is almost horizontal in our OSC. We further discuss the influence of the optical cavity on the effective radiative exciton decay rate and conclude by simulating cathode material-dependent contour plots of the expected forward luminance for the fabricated LEC stacks. The measured electrode-dependent transients can be replicated qualitatively by these simulations, which shows that the significant electrode-dependent luminance differences are explained by both the optical cavity properties and the transients of the exciton generation zones.

Thereby, we present evidence that the LEC performance, in contrast to the common conception, is dependent on the electrode material selection and that a rational LEC design should thus collectively consider the electrode material properties, the active-material thickness, and its composition.

## Experimental Section

### Ink Fabrication

The active material comprises a blend of an electroluminescent conjugated polymer, a phenyl-substituted poly(*para*-phenylenevinylene) copolymer termed “Super Yellow” (SY, Livilux PDY-132, Merck, GER), a hydroxyl end-capped trimethylolpropane ethoxylate (TMPE–OH, *M*_n_ = 450 g mol^–1^, Sigma-Aldrich, USA) ion transporter, and a KCF_3_SO_3_ (Sigma-Aldrich, USA) salt. The salt (ion transporter) is dried in a vacuum oven at *p* < 10^2^ Pa and 190 °C (50 °C) for 12 h before use. The active-material constituents are separately dissolved in cyclohexanone (Sigma-Aldrich, USA) in a concentration of 10–15 g L^–1^ (SY), 10 g L^–1^ (TMPE–OH), and 10 g L^–1^ (KCF_3_SO_3_). These master inks are blended in a solute mass ratio of SY/TMPE–OH/KCF_3_SO_3_ = 1:0.1:0.03 for the formulation of the active-material ink, which is stirred for ≥24 h at 70 °C in a glovebox ([O_2_] < 1 ppm, [H_2_O] < 1 ppm).

### Device Fabrication

The indium tin oxide (ITO) coated glass substrates (ITO thickness = 145 nm, substrate area = 30 × 30 mm^2^, substrate thickness = 0.7 mm, Thin Film Devices, USA) are cleaned by sequential ultrasonication in a detergent (Extran MA 01, Merck, GER), deionized water, acetone (VWR, GER), and isopropanol (VWR, GER) before being dried in an oven at 120 °C for ≥12 h. The active-material ink is spin-coated on the ITO substrate at 1000–4500 rpm for 120 s and dried on a hot plate at 70 °C for 1 h. The active-material thickness (*d*_AM_) is measured with a stylus profilometer (Dektak XT, Bruker, USA). Depending on the SY concentration and spinning parameters, we yield *d*_AM_ of (100 ± 5) nm, (180 ± 5) nm, and (320/340/330 ± 5) nm for the (Al/Ag/Ca) cathodes, respectively. The Al, Ag, and Ca reflective top cathodes are deposited by thermal evaporation at *p* < 2 × 10^–4^ Pa, with a shadow mask defining the cathode area. The spatial overlap between the cathode and the anode defines four 2 × 2 mm^2^ LEC pixels on each substrate. The LECs are encapsulated with a cover glass (24 × 24 mm^2^, VWR, GER) using a UV-curable epoxy resin (Ossila, UK) and measured under ambient conditions.

### Device Characterization

The current–voltage measurements are performed using a computer-controlled source measure unit (SMU 2400, Keithley, USA). The devices are driven by a current density of 25 mA cm^–2^, using a voltage compliance of 21 V. All devices are biased with ITO as the positive anode. The nonpolarized, angle-resolved emission spectra and intensity are measured using a custom-built, calibrated spectro-goniometer. The device is placed in a sample holder, which aligns the emission area of the device with the rotation axis of a stepper motor. A fraction of the emitted light is collected by a collimating lens (ϕ = 7.2 mm, F230 SMA-A, Thorlabs, Germany) positioned 75 mm away from the device. This results in a small and constant solid collection angle (Ω) of 0.007 sr. An optical fiber delivers the collimated light to a CCD-array spectrometer (Flame-S, OceanOptics, USA, linearity >99%, optical resolution FWHM <5 nm). By a rotation of the sample, as controlled by a Python-based virtual instrument, the viewing angle is varied between −80° to +80° in steps of 5° or 10°. The forward luminance is derived from the 0° measurement. A schematic of the setup is depicted in ref (15), Figure 1. In total, 40 independent devices are measured, cf. Supporting Information Section 4, and the presented data is chosen from representative devices within this set.

The photoluminescence (PL) spectra are collected with a commercial PL quantum yield setup (C9920, Hamamatsu Photonics, JP) with the sample placed in an integrating sphere. The sample is excited by a 150 W xenon lamp equipped with a monochromator. The excitation wavelength is set to 450 nm, and the PL signal is recorded by a CCD spectrometer.

### Modeling

All simulations are performed with the commercial software Setfos (Version 5.2, Fluxim AG, Switzerland). The model of the device stack comprises the following layers with thicknesses matching the experimental specifications (if not stated otherwise as in Figure 3):

Air (inf.)/reflective top electrode (100 nm of Al, Ag, or Ca/Al)/SY (100, 180, 320, 330, 340 nm)/ITO (145 nm)/Glass (0.75 mm)/Air (inf.).

The CEG in the active material (Figure 2) is derived by finding the absolute minimum mean square error (MSE) between the measured radiant intensity *I*_θ,λ_^meas^ and the simulated radiant intensity *I*_θ,λ_^sim^ for a set of *N*_λ_ wavelengths λ and *N*_θ_ viewing angles θ.
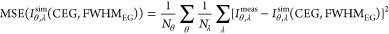
3

The optical model uses a predefined set of exciton generation profiles *G*(*x*), each represented by a Gaussian distribution of full width at half-maximum FWHM_EG_ peaking at CEG, to generate the simulated spectral output *I*_θ,λ_^sim^(CEG, FWHM_EG_) at a given *d*_AM_. The assessable peak values for *G*(*x*) range between 0.05 and 0.95 (corresponding to the relative interelectrode distance with *x* = 0.0 corresponding to the anode position and *x* = 1.0 to the cathode position) using a grid step size of 0.01. The assessable FWHM_EG_ values for *G*(*x*) range from 0.01 (minimal step size, effectively emulating a delta distribution), 0.1, 0.2, ... in steps of 0.1 to 1.0.

Since a narrow *G*(*x*) (FWHM_EG_ < 10% of *d*_AM_) is mostly found as the best fit (except for the *d*_AM_ = 330 nm devices shortly after the turn-on), we use a delta distribution to approximate the outcoupling landscape and SPP analysis (Figures 3 and 5) for reduced computational effort. For the analysis of cavity influences on an extended emission zone (Figure 4), we set *G*(*x*) as a second-order super-Gaussian centered at *x* = 0.5 and FWHM_EG_ = 20% of *d*_AM_.

The average dipole orientation is set to *a* = 0.05.^39^ The emission spectrum of SY is set equal to the measured PL spectrum of a 17 nm thin film of Super Yellow. We set 0.6 as the photoluminescent quantum yield and 2 ns as the natural exciton lifetime in the film, corresponding to *k*_nr_ = 2 × 10^8^ s^–1^ and *k*_r,0_ = 3 × 10^8^ s^–1^.^15,48^ The optical constants (*n* and *k* values) of the active film are taken from ref (44) and the optical constants of the electrode materials are taken from the Setfos database for SY.

### Confidence Interval Calculation

The error bands σ_tot_^±^(*t*) of CEG(*t*) in Figure 2 are calculated as the Euclidean norm of two contributing error dimensions at a given point in time *t.*
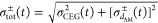
4

First, we calculate σ_CEG_, the standard error of the best-fitting CEG, from the Covariance matrix of the function MSE(*x*_1_ = CEG, *x*_2_ = FWHM_EG_) at the point of optimized parameters ζ. The respective matrix element (1,1) reads
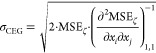
5and quantifies the inverse curvature of the MSE landscape.^53,54^ A shallow error landscape is thus translated into a high standard error, while a sharp minimum gives a low error. Here, we interpret every spectro-goniometer sweep collecting one spectrum with *N*_λ_ wavelength bins for *N*_θ_ angles as one single independent measurement (*N* = 1). Second, the film thickness *d*_AM_ used for the simulation is varied by ±5 nm around the experimentally determined value. The resulting shift for the best-fitting CEG is taken as the error , which may differ in both directions (±).
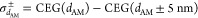
6

## Data Availability

All relevant experimental data and the Setfos simulation files are available for download here 10.6084/m9.figshare.27248310.v1.
