# Supplementary material for: Impact of the Electrode Material on the Performance of Light-Emitting Electrochemical Cells
Source: ACS Appl Mater Interfaces. 2025 Jan 10;17(3):5184–92. doi: 10.1021/acsami.4c18009 (PMC11758773; doi:10.1021/acsami.4c18009)
Supplement: Supplementary file 1 — am4c18009_si_001.pdf [file am4c18009_si_001.pdf]

Supporting Information

Impact of the electrode material on the performance of light-emitting electrochemical cells

Anton Kirch[1,#], So-Ra Park[1,#], Joan Ràfols-Ribé[1,2], Johannes A. Kassel[3], Xiaoying Zhang[1], Shi Tang[1,2], Christian Larsen[1,2] and Ludvig Edman[1,2,4]*

[1] The Organic Photonics and Electronics Group, Department of Physics, Umeå University, SE-90187 Umeå, Sweden

[2] LunaLEC AB, Umeå University, SE-90187 Umeå, Sweden

[3] Max Planck Institute for the Physics of Complex Systems, Nöthnitzer Straße 38, 01187 Dresden, Germany

[4] Wallenberg Initiative Materials Science for Sustainability, Department of Physics, Umeå University, SE-90187 Umeå, Sweden

[#] These authors contributed equally.

*E-mail: ludvig.edman@umu.se

Contents

1. Experimental data after LEC turn-on 2
2. The number of ions in EDLs 3
3. Influence of the emitter orientation on SPP coupling 4
4. Experimental data of all investigated devices 5

1. Experimental data after LEC turn-on

To illustrate the turn-on behavior of the investigated LECs, we plot the same data as in Figure 1, main manuscript, for the first 1000 s. The turn-on characteristics of a functional device are a decreasing driving voltage and increasing luminance during the initial operation. Note that the devices are driven at a constant current density of 25 mA cm^{-2} while setting a voltage compliance of 21 V.

The forward luminance is derived from the 0° spectro-goniometer measurement. The luminance monitoring was accidentally not always started right after the current supply, as the turn-on monitoring is not the scope of this work.

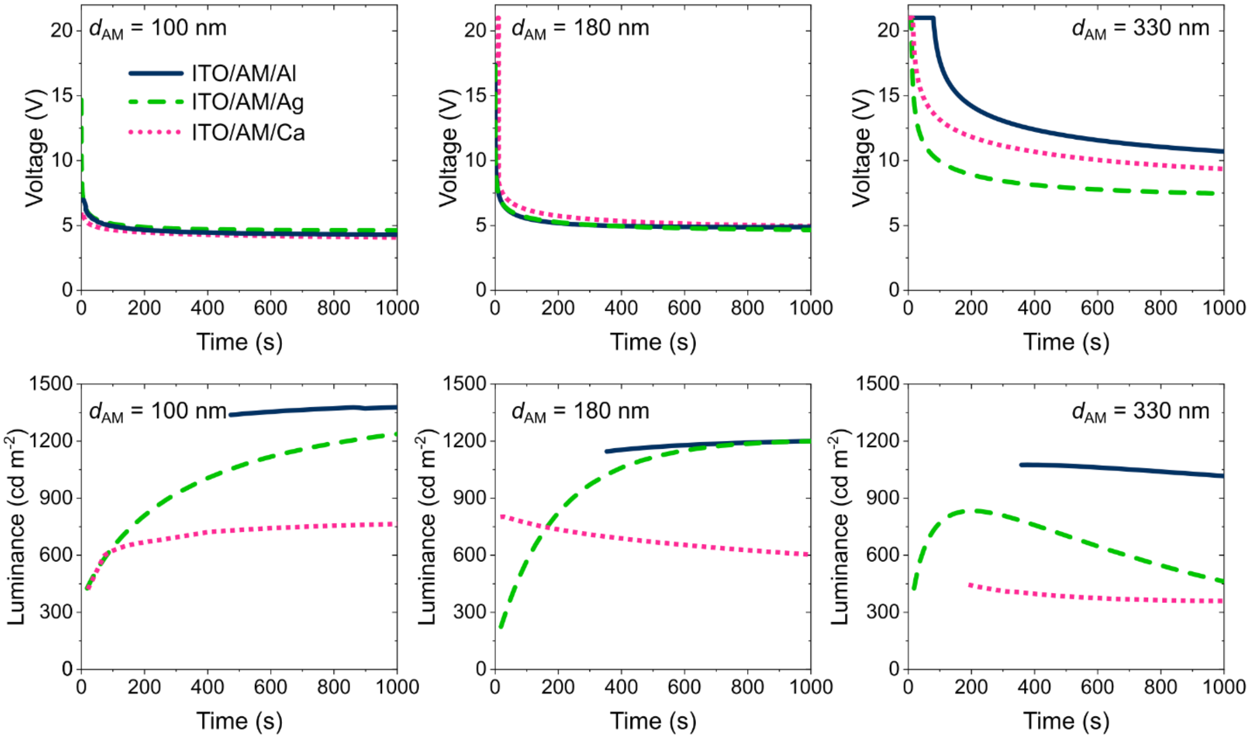

Figure S1. LEC characterization (same data as in Figure 1, main manuscript) for the first 1000 s.

2. The number of ions in EDLs

The number of non-compensated ions "locked up" in an EDL ($n_{\text{ion,EDL}}$) is equal to the net electronic charge on the electrode side of the EDL, and $n_{\text{ion,EDL}}$ can be calculated by:

$$C_{\text{EDL}} = \frac{Q_{\text{EDL}}}{V_{\text{EDL}}} = \frac{e \cdot n_{\text{ion,EDL}}}{V_{\text{EDL}}} = \varepsilon_{\text{r}} \cdot \varepsilon_0 \cdot \frac{A_{\text{EDL}}}{d_{\text{EDL}}} \quad \text{(S1)}$$

$$n_{\text{ion,EDL}} = \frac{\varepsilon_{\text{r}} \cdot \varepsilon_0 \cdot A_{\text{EDL}} \cdot V_{\text{EDL}}}{e \cdot d_{\text{EDL}}} \quad \text{(S2)}$$

The variables and parameters in the above equation, with our employed values in parenthesis, are as follows: C_{EDL} is the capacitance of the EDL, Q_{EDL} is the net charge on either side of the EDL, V_{EDL} is the voltage drop over the EDL, e is the elementary charge, ε_{r} (≈ 3) is the relative permittivity of the active material, ε_0 is the vacuum permittivity, A_{EDL} is the effective cross-section area of the EDL and it is estimated to be approximately equal to the cross-section area of the device ($A_{\text{EDL}} \approx A_{\text{AM}} = 4 \cdot 10^{-6}$ m^2), d_{EDL} (≈ $0.5 \cdot 10^{-9}$ m) is the effective thickness of the EDL. If we assume that ohmic injection is attained when V_{EDL} exactly compensates the injection barrier at the electrode/active-material (AM) interface, we get that V_{EDL} (Al/AM) = 1.7 V, V_{EDL} (Ag/AM) = 1.7 V, V_{EDL} (Ca/AM) = 0.3 V, and V_{EDL} (ITO/AM) = 0.4 V. By plugging these values into Eq. (2), we find that $n_{\text{ion,EDL}}$ (Al/AM) = $n_{\text{ion,EDL}}$ (Ag/AM) = $2.3 \cdot 10^{12}$ ions, $n_{\text{ion,EDL}}$ (Ca/AM) = $0.4 \cdot 10^{12}$ ions, and that $n_{\text{ion,EDL}}$ (ITO/AM) = $0.5 \cdot 10^{12}$ ions.

These numbers can be compared to the *total* number of ions in the active material, which can be calculated with the following equation:

$$n_{\text{ion,AM}} = A_{\text{AM}} \cdot d_{\text{AM}} \cdot \frac{m_{\text{salt}}}{m_{\text{AM}}} \cdot \rho_{\text{AM}} \cdot \frac{1}{M_{\text{salt}}} \cdot N_{\text{A}} \cdot 2 \quad \text{(S3)}$$

Here, A_{AM} (= $4 \cdot 10^{-6}$ m^2) is the effective cross-sectional (or emission) area of the LEC, d_{AM} (= 100 or $180 \cdot 10^{-9}$ m) is the thickness of the active material, $\frac{m_{\text{salt}}}{m_{\text{AM}}}$ (=$\frac{0.03}{1.13}$) is the mass fraction of the KCF_3SO_3 salt in the active material, ρ_{AM} (≈ $1 \cdot 10^{6}$ g m^{-3}) is the density of the active material, M_{salt} (= 188.17 g mol^{-1}) is the molar mass of the salt, N_{A} is Avogadro's constant, while the final factor "2" converts the number of salt molecules to the number of ions.

This results in that the active material contains a total of $n_{\text{ion,AM}}$ = $6.8/12.2 \cdot 10^{13}$ ions, divided into $3.4/6.1 \cdot 10^{13}$ K^+ cations and $3.4/6.1 \cdot 10^{13}$ $CF_3SO_3^-$ anions.

In other words, 1.5/0.8 % of the $CF_3SO_3^-$ anions in the active material are "lost" to the anodic EDL at the ITO electrode in all three investigated LECs. In contrast, 6.7/3.7 % of the K^+ cations are "lost" at the EDL at the Al and Ag cathodes, while 1.1/0.7 % of the K^+ cations are lost at the EDL at the Ca cathode. Since it is the remaining ions, not "lost" to the EDLs that can contribute to the electrochemical doping, this suggests that the maximum attainable p-type doping concentration varies.

3. Influence of the emitter orientation on SPP coupling

The impact of SPP coupling on the effective radiative exciton decay rate $k_r^*(x)$ varies significantly with the emissive dipole orientation coefficient *a*. Figure S2 shows $k_r^*(x)$ for the investigated LEC configurations depending on *a*. The case *a* = 0.05 is presented in the main manuscript, Figure 4. A small *a* indicates preferably horizontal dipole alignment (as for the material Super Yellow used in this study, *a* = 0.05), *a* = 1/3 denotes isotropic (random) dipole orientation, and *a* = 1 means exclusively vertical dipole orientation. The more vertical the mean dipole orientation (*a* is closer to 1), the more the radiative coupling of excitons to SPP modes is enhanced (e.g. S. Nowy et al., *Journal of Applied Physics* **2008**, *104* (12), 123109).

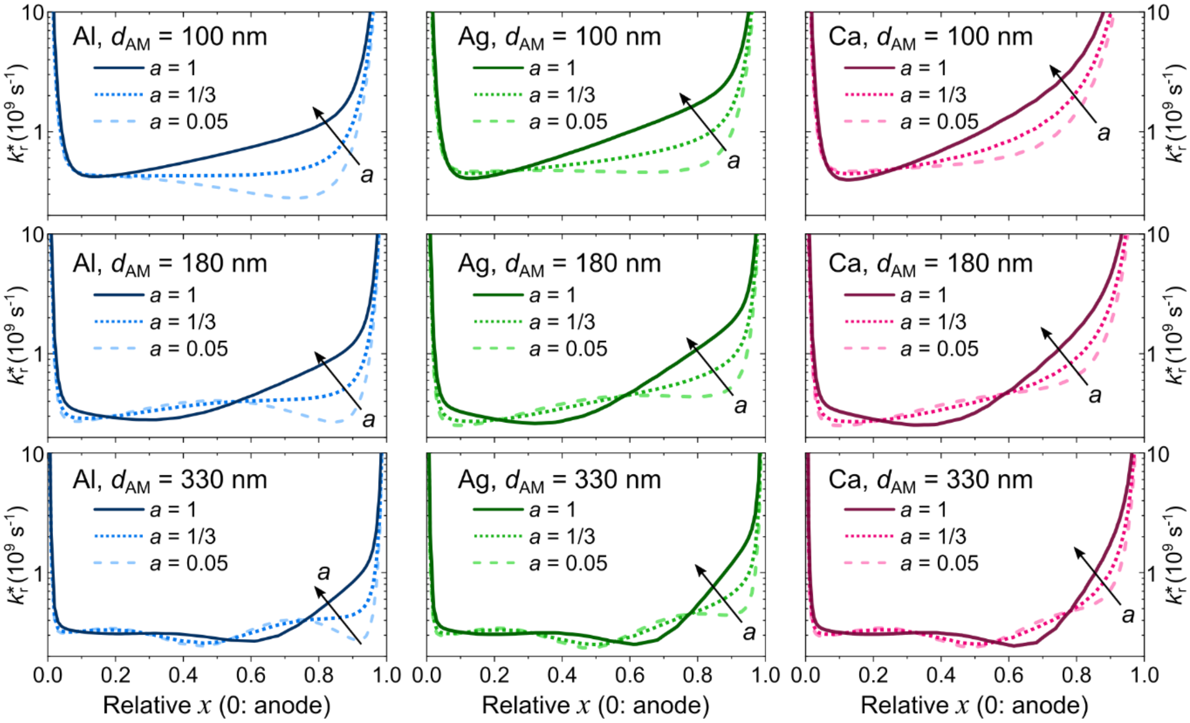

Figure S2. The impact of the emitter dipole orientation *a* on the effective radiative exciton decay rate $k_r^*(x)$ for the investigated LEC configurations. In the main manuscript, the data for *a* = 0.05 is presented.

4. Experimental data of all investigated devices

The following Figures S3-S5 show the data of all investigated devices. The data presented in the main manuscript, Figure 1, and in Figure S1 are highlighted with an asterisk (*). If several devices are measured for one LEC configuration, a representative device was chosen or the one that was investigated long enough according to the final data analysis. Note that here the CEG is calculated with a delta-distributed exciton generation profile $G(x)$, which induces errors for the thick-film LECs (perceivable as jumps in Figure S5). Concerning the data selected for the main manuscript (*), the CEG was determined again more accurately using a Gaussian-shaped exciton generation profile with varying thickness $\mathrm{FWHM}_{\mathrm{EG}}$ and peak position CEG.

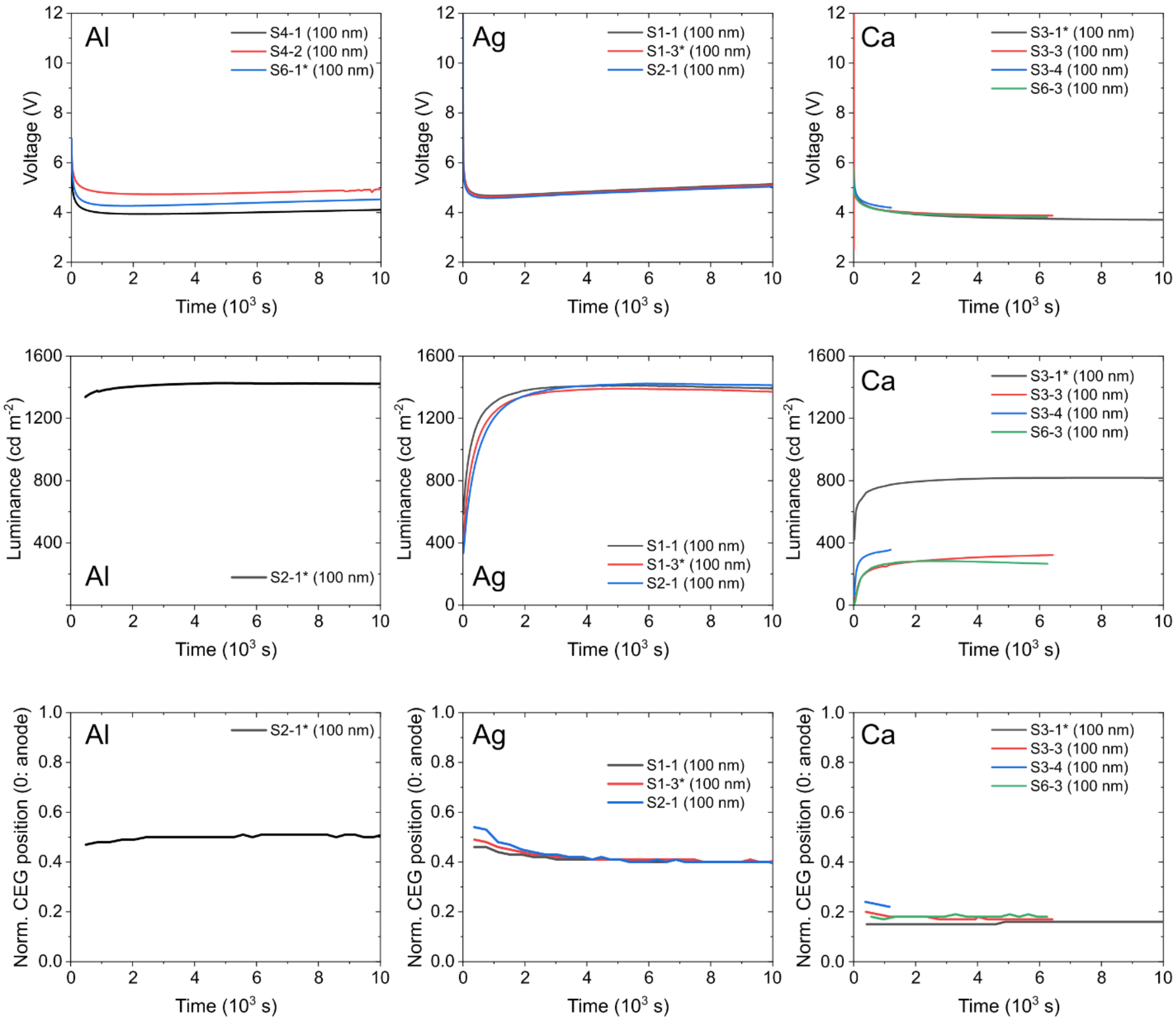

Figure S3. All experimental data for devices with d_{AM} = 100 nm.

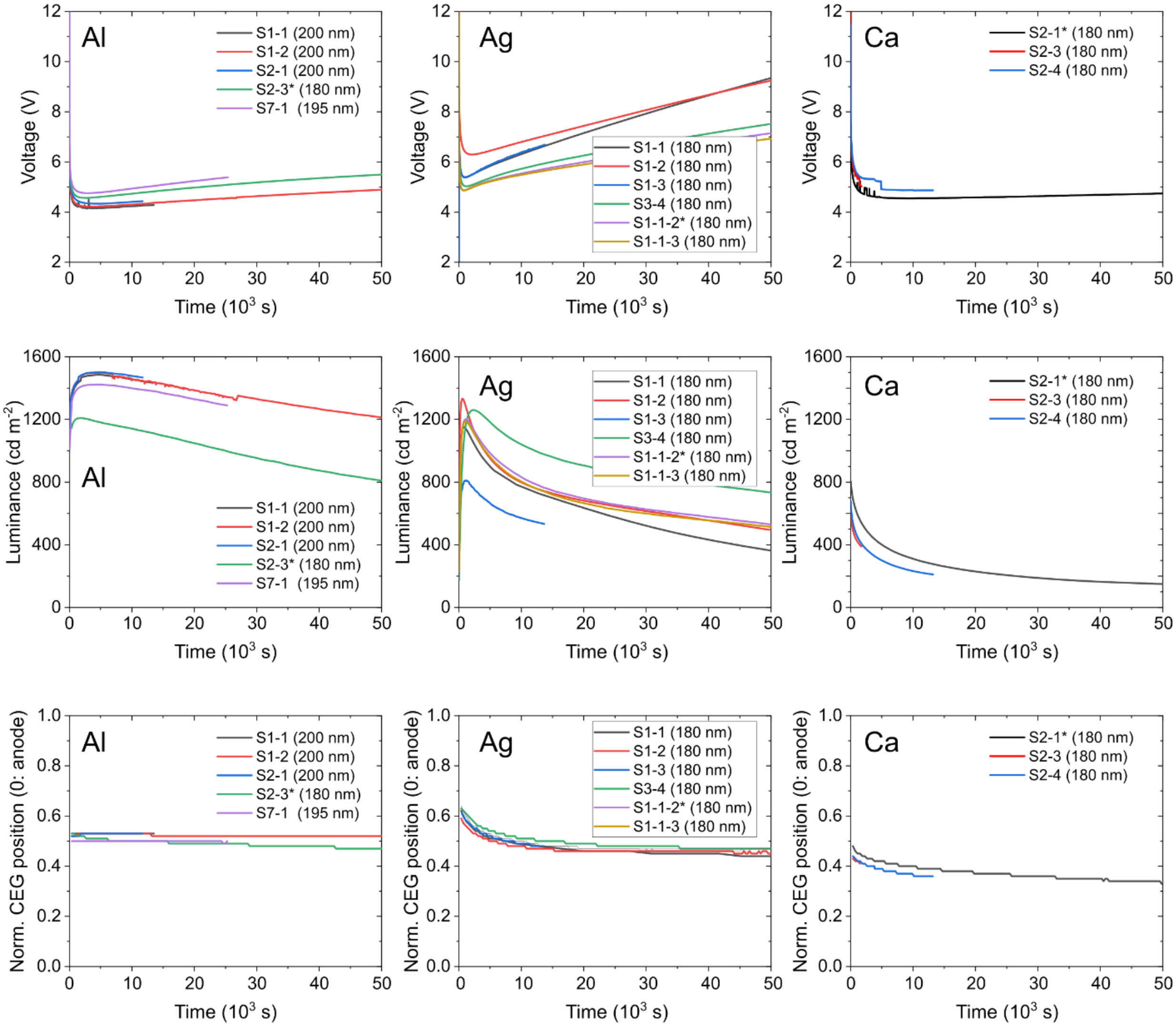

Figure S4. All experimental data for devices with $d_{AM} \approx 180$ nm.

The jumps in the calculated CEG position for thick devices, Figure S5, are an artifact of using a delta-distributed exciton generation profile $G(x)$ to reduce computation time for initial screening. For the data set selected for the main manuscript, the width of $G(x)$ was set as a parameter.

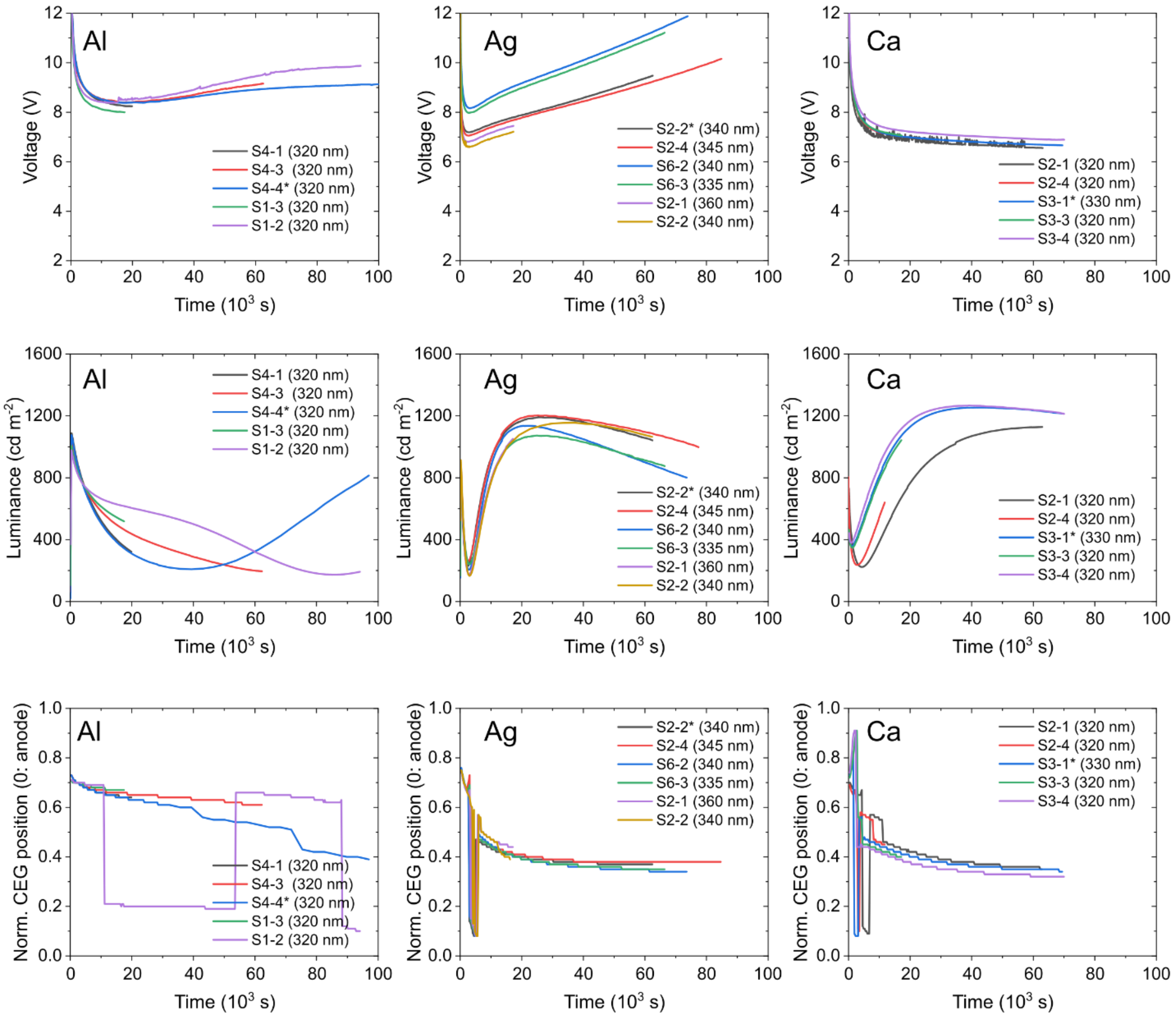

Figure S5. All experimental data for devices with $d_{AM} \approx 330$ nm.
